# Brain–Computer Interfaces with Intracortical Implants for Motor and Communication Functions Compensation: Review of Recent Developments

**DOI:** 10.17691/stm2024.16.1.08

**Published:** 2024-02-28

**Authors:** O.A. Mokienko

**Affiliations:** Senior Researcher, Mathematical Neurobiology of Learning Laboratory; Institute of Higher Nervous Activity and Neurophysiology of Russian Academy of Sciences, 5a Butlerova St., Moscow, 117485, Russia; Senior Researcher, Engineering Center; N.I. Pirogov Russian National Research Medical University, 1 Ostrovityanova St., Moscow, 117997, Russia; Researcher, Brain–Computer Interface Group of Institute for Neurorehabilitation and Restorative Technologies; Research Center of Neurology, 80 Volokolamskoye Shosse, Moscow, 125367, Russia

**Keywords:** brain–computer interface, neural implant, tetraplegia, locked-in syndrome, anarthria

## Abstract

Brain–computer interfaces allow the exchange of data between the brain and an external device, bypassing the muscular system. Clinical studies of invasive brain–computer interface technologies have been conducted for over 20 years. During this time, there has been a continuous improvement of approaches to neuronal signal processing in order to improve the quality of control of external devices. Currently, brain–computer interfaces with intracortical implants allow completely paralyzed patients to control robotic limbs for self-service, use a computer or a tablet, type text, and reproduce speech at an optimal speed. Studies of invasive brain–computer interfaces regularly provide new fundamental data on functioning of the central nervous system. In recent years, breakthrough discoveries and achievements have been annually made in this sphere.

This review analyzes the results of clinical experiments of brain–computer interfaces with intracortical implants, provides information on the stages of this technology development, its main discoveries and achievements.

## Introduction

Brain–computer interfaces (BCIs, neural interfaces) allow direct information exchange between the brain and the computer with the data transfer to an external technical device. Such interfaces include electrodes to record brain activity signals, a signal processing system (filtering, feature extraction, decoding, classification and conversion into a control command), as well as a controlled external technical device [1]. In case of invasive BCIs, the system can also send a signal in the opposite direction: from external sensors to neural implants in the cerebral cortex, thus ensuring neuromodulation (see the Figure) [2].

**Figure:**
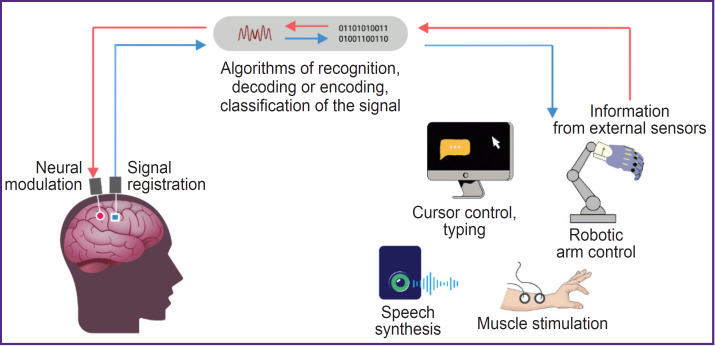
Invasive brain–computer interface: general scheme and technology capabilities

In recent years, an extensive evidence base on the use of non-invasive BCIs in rehabilitation after stroke has been formed [3–13]. Signal recording in such interfaces is conducted from the head surface, most often using electroencephalographic (EEG) sensors or near-infrared spectroscopy (NIRS) during motor imagery practice [14, 15]. Non-invasive BCIs allow motor imagery practice to stimulate neuroplasticity and restore motor function provided that the patient has rehabilitation potential [10–13].

The signals recorded in invasive BCIs are the local field potential (using extracortical or intracortical sensors) and neurons spiking activity (using intracortical sensors) [16]. Despite high cost of development and research, as well as the need for surgical intervention, invasive BCIs are no-compromise means of interaction with the environment for completely paralyzed and speechless patients, who preserve cognitive functions in case of tetraplegia and anarthria or the locked-in syndrome of various etiologies. Unlike non-invasive BCIs, the evidence base for which includes multiple randomized clinical trials and their meta-analyses, the clinical application of invasive neural interfaces is limited to several dozen cases only. However, almost each new case is a scientific breakthrough, and the corresponding articles are published in the top-rated journals [17–27].

Currently, low or minimally invasive BCIs with extracortical [17, 26, 28, 29] or endovascular [30, 31] sensors are being developed; but advances in development of neural interfaces with intracortical sensors [2, 32] are of greater scientific interest. The obvious advantages of such BCIs include the following: 1) getting signals of brain activity with the highest temporal and spatial resolution; 2) high signal-to-noise ratio; 3) the closest or the most precise placement of electrodes in the target brain areas; 4) transmission of the signal in the opposite direction — from external sensors to the cerebral cortex [2, 32]. Due to high, one-neuron only, spatial resolution of signal recording or neuromodulation, intracortical neural implants allow to obtain new data on localization of specific functions in the cerebral cortex and the characteristics of its neurons functioning [16, 24, 25, 33, 34].

**The aim of this review** is to analyze and describe the capacities of brain–computer interfaces with intracortical implants in rehabilitation of patients with severe motor disorders.

## Literature search methodology

Literature search was conducted in the MEDLINE (PubMed) database using the search query: ((invasive[tiab] OR intracortical[tiab]) AND (brain-computer[tiab] OR brain-machine[tiab] OR “neural interface*”[tiab]) OR intracortical implant*[tiab]) AND humans[mh]. Additionally, a literature search was conducted in the eLIBRARY.RU system using the keywords: “brain–computer interface”, “neural computer interface”, “neural interface”. The date of search was July 15, 2023.

Articles were selected for analysis based on the following criteria: 1) articles or letters to the editor published in the peer-reviewed scientific journals; 2) publications on the use of invasive BCIs with intracortical sensors in humans; 3) articles on using BCIs to compensate for motor or speech dysfunctions.

## Preclinical studies and first experiments

Studies on the use of implanted sensors to record signals from the monkey cerebral cortex started in the 1960s [35, 36]. In the 1970s, in experiments with monkeys, one managed to create a system for converting cortical signals into cursor movement in real time [37, 38]. The animals were able to control the cursor by modulating signals from the motor cortex, even without an actual movement. In the late 1990s and early 2000s, researchers of preclinical studies applied BCI systems with robotic limbs as an externally controlled device [39–47]. With these technologies, animals could feed themselves. After the start of clinical experiments of invasive BCIs, animal studies continue to test several scientific hypotheses and search for new approaches to signal processing [48–57].

It is believed that the first experiment related to control of an external device by signals from a neural implant in the human brain was conducted in 1963 by British neurosurgeon Grey Walter [58, 59]. The researcher wanted to test the hypothesis that the intention to perform an action is accompanied by certain bursts in neuronal activity. Patients, who previously had electrodes implanted in the motor cortex of their brain for medical reasons, were asked to switch projector slides by pressing a button. Here, the button was dummy, though the patients were not aware of that. In fact, the slides were switched by an amplified signal from the neuroimplant. The patients were surprised that the slide projector anticipated their actions.

## First clinical studies, neurotrophic electrode

In the late 1990s, researchers led by P.R. Kennedy were the first to implant neurotrophic electrodes into several patients with tetraplegia for long-term recording of cortical signals [60–62]. The electrodes consisted of two insulated gold wires inside a glass cone of 1.5 mm long and 0.1–0.4 mm in diameter having autologous neurotrophic factors. This was a wireless system [63]. 1.5–3 months after implantation, the neuron processes in the cerebral cortex grew into the tip of the electrode with subsequent myelination. A few weeks after implantation, the first signals could be recorded, and within 1.5–3 months the signal became stable. Using neurotrophic electrodes, one managed to record a signal that remained stable for at least four years [64].

The first implantation of such a neurotrophic electrode was performed in a woman at the late stage of amyotrophic lateral sclerosis (ALS) [60]. Localization of the implantation site, the hand representation area in the right motor cortex, was determined using the functional magnetic resonance imaging (fMRI) during the imagination of hand movements and speech articulation. Almost immediately after the neuronal activity signal stabilization, the patient learned to control the cursor in the vertical direction. The implant had been functioning for 76 days — until the last days of the patient’s life. Then, the neurotrophic electrodes were implanted into other patients with tetraplegia and anarthria: in 1998, into a 53-year-old man 3 months after he had a brainstem stroke [61, 62], in 1999, into a 40-year-old man with a 12-year history of progressive mitochondrial myopathy [62], and in 2004, into a 26-year-old man who had a 5-year brainstem stroke [64, 65]. Using the invasive interface, the first of these patients managed to control the cursor in various directions on the display, could click the target and control the fingers of a virtual hand. The most interesting observation for the researchers was the ability to control the cursor without the need to imagine movements or any other standard brain paradigms: the patient controlled the cursor by self-will. Researchers associated this phenomenon with neuroplasticity, and called the electrode implantation area the “cursor cortex” [61]. However, the patient needed six months to master the typing skill after stabilization of the recorded signal, and the typing speed was three characters per minute. At that time, it was already possible to type text with the same speed using non-invasive BCIs.

During the experiment, the patient with mitochondrial myopathy developed a severe cognitive impairment due to disease progression, but was able to control the cursor in one direction [62].

In 2004, in order to interpret speech-associated neural activity, a neurotrophic electrode was implanted into a 26-year-old patient with brainstem stroke [64, 65]. The implant was grafted into a region of the cortex involved in planning the sound articulation. In this BCI with a Kalman filter decoder, neural signals generated during speech attempts were used to control a speech synthesizer. The accuracy of vowel sound reproduction by the patient after 25 training sessions was 70%.

Thus, the studies led by P.R. Kennedy developed a methodology to identify a cortical area for sensor implantation in patients with plegia and anarthria. For the first time, recording electrodes were implanted into the human cerebral cortex for a long period of time, as well as the researchers demonstrated: the electrodes safety and feasibility for using to control a cursor, a hand avatar, and a speech synthesizer through voluntary modulation of cortical signals even years after the plegia onset. Despite the limited functionality of the first invasive BCIs, in the late 1990s the possibility of creating alternative means of communication and self-care for patients with tetraplegia was shown [60–62, 64, 65].

## Early research under the BrainGate project

In the early 2000s, the BrainGate project launched a series of clinical studies of invasive BCIs. The neuroimplant was an array of 100 silicon microelectrodes (96 active), 1.5 mm long, arranged in a 10×10 pattern on a 4×4 mm platform (Blackrock Microsystems, Salt Lake City, Utah, USA) — the so-called “Utah array” [66]. Before that, the microimplant had been studied in preclinical studies [67–70].

The first patients to whom BrainGate sensors were implanted in 2004–2005 were 25 and 55 year old men with spinal cord injury (SCI) at the level of the fourth cervical vertebra (C4 ASIA A in line with the American Spinal Injury Association scale), which had been occurred 3 and 5 years before implantation, respectively [18, 71]. Researchers led by J.P. Donoghue demonstrated the capacity of this sensor to record both neurons spiking activity and local field potentials during 6.5 and 11 months (for the first and second patient, respectively), as well as the patients’ ability to control these signals years after the corticospinal tract breakage. Neural decoding in the BCI circuit allowed to open e-mail and control the TV using the “neural cursor”. The first patient successfully reached 73–95% of targets with the “neural cursor”; the average time to reach the target was 2.5 s [18, 71].

In further studies involving patients with brainstem stroke or ALS, signal processing algorithms were optimized, which allowed to reduce calibration time and to get a better cursor control [72–74].

Later, it was shown that even 1000 days (2.7 years) after implantation, the microelectrode array continued recording the signals: the patient demonstrated consistently high levels of control quality over five consecutive days of the experiment. The rate of successful target achievement in this experiment was

94.9% for the radial and 91.9% for the random target location (which exactly simulates the computer mouse). Of the 564 tasks, only 37 failed by timeout, but not due to cursor navigation errors [75]. These results took down the investigators’ concerns about the risk of fast decline in the electrodes function due to tissue reaction to the implant. By now, the even longer duration of functioning have been shown for neural implants [19].

## Robotic arm control

Subsequent studies were mainly related to control of an external robotic multi-joint arm to do functionally significant movements (Table 1). This type of task involved manipulating an object in three dimensions along curved trajectory and sequential actions at different joints. Compared to cursor control, one needs more precise control of speed and movement pattern, as well as exact positioning and command planning.

**Table 1:** Main results of clinical studies of neural interfaces with a robotic arm

References	Patients	BCI features	Results
Hochberg et al., 2006 [18]	M., 25 years, SCI C4 ASIA A which occurred 3 years before the experiment (MN), 1–9 months after implantation (T2)	1 implant in M1, a linear filter, a robotic arm with elementary movements	Control of the robotic arm: elementary moves with one degree of freedom
Hochberg et al., 2012 [19]	1) F., 58 years, brainstem stroke which occurred 15 years before the experiment, 1952–1975 days after implantation (S3); 2) M., 66 years, brainstem stroke which occurred 5.5 years before the experiment, 166 days after implantation (T2)	1 implant in M1, 2 types of the robotic arm	S3: touching the target object in 76%, grasping in 47% of attempts, drinking coffee using the robotic arm — 4 out of 6 attempts; T2: touching the target object in 96%, grasping in 62% of attempts Average task completion time: 8.5 s
Collinger et al., 2013 [20]	F., 52 years, SCA, diagnosis set 13 years before the experiment, 10–98 days after implantation	2 implants in the anterior central gyrus, a robotic arm with 7 degrees of freedom	Control of the robotic arm with 7 degrees of freedom with a success rate of 91.6%; 15–17 scores for the ARAT test completion using the robotic arm
Wodlinger et al., 2015 [76]	F., 52 years, SCA, 119–280 days after implantation	2 implants in the anterior central gyrus, a robotic arm with 10 degrees of freedom	Control of the robotic arm with 10 degrees of freedom; 12–17 scores for the ARAT test completion using the robotic arm
Aflalo et al., 2015 [25]	M., 32 years, SCI C3–C4 which occurred 10 years before the experiment, 16 days–21 months after implantation	2 implants in the posterior parietal cortex (imagining the plan for the arm reaching and object grasping), robotic arm with 17 degrees of freedom	The ability to control robotic limbs with a signal having its source in the posterior parietal cortex was demonstrated
Downey et al., 2017 [77]	1) F., 55 years, SCA, 795–850 days after implantation; 2) M., 30 years, SCI C5–C6 ASIA B, 661–673 days after implantation	2 implants in the anterior central gyrus, a robotic arm; 2 implants in the anterior central and 2 — in the posterior central gyrus (were not used)	Increased BCI performance when manipulating with the robotic arm due to task optimization
Flesher et al., 2021 [23]	M., 29 years, SCI C5–C6 ASIA B which occurred 10 years before the experiment	Bidirectional BCI: 2 implants in the anterior central gyrus and 2 in the posterior central gyrus, tactile sensors in the robotic hand (touch and pressure force)	With tactile feedback, the ARAT score increased from 17 to 21, the average test execution time decreased from 21.0 to 10.2 s
Handelman et al., 2022 [78]	M., 49 years, SCI C5 ASIA B which occurred 30 years before the experiment	Bimanual BCI with 6 implants: 2 implants in each of the anterior central gyrus and posterior central gyrus of the dominant hemisphere; 1 implant in each of the anterior central gyrus and posterior central gyrus, 2 robotic arms, semi-autonomous system	85% success rate in bimanual task (eating with a fork and a knife)

Note: SCI — spinal cord injury; SCA — spinocerebellar ataxia; MN, S3, T2 — patient IDs in the BrainGate project; M./F. — patient’s gender; M1 — primary motor cortex.

In the experiment of 2012, two patients with tetraplegia controlled a multi-joint robotic arm and managed to reach out and grasp the target object in 47 and 62% of attempts [19]. By controlling a robotic arm, the experiment participant was able to bring a glass of coffee to her mouth and drink it with a straw. Owing to the BCI, she had performed a self-care act for the first time in 15 years. Despite the 5-year implantation period, the electrode array recorded signals sufficient to control the robot, but a decrease in their amplitude and a drop-down in active electrode channels were observed.

In another study [20], a patient with spinocerebellar ataxia had two sensors implanted in the cortical area representing finger and forearm muscles of the dominant hemisphere. The anthropomorphic robotic arm design allowed movements with seven degrees of freedom. Already on the second day of training, the patient was able to freely operate the robotic arm in three dimensions. Over the next 13 weeks, the control quality consistently improved. The movements were smooth and coordinated, at a speed close to the same of a healthy person’s arm. The robot control level allowed to perform subtle adjustments and manipulations with various balls, cubes, and sticks. On average, the targets were successfully achieved in 92% of attempts, and in the ARAT test (Action Research Arm Test — a test to assess arm movements in central paresis) 17 out of 27 scores were received with the robotic arm.

In subsequent clinical experiments, researchers improved the BCI system architecture and signal processing algorithms, which allowed to control a robotic arm with 10 degrees of freedom [76], improve the grasp quality [77, 79], and reduce the system calibration time from 10 to 3 min [80]. Moreover, additional cortical areas for electrode implantation were identified as sources of signals that were associated with movement planning [25].

In one of recent studies, the BCI design included two robotic arms to perform complex bimanual tasks [78]. To control this system, a 30-years-aged patient with SCI was implanted with six electrode arrays in both brain hemispheres. A semi-automatic system was used to self-feed manipulating a fork and knife: some specific movements were programmed, and some were controlled by brain signals. The patient successfully completed 85% of bimanual tasks.

## Brain–computer interface with electrical stimulation of paralyzed muscles

Electrical stimulation of patient’s muscles by the modulation of motor cortical signals ensures a more natural reaction of the motor system [81]. By now, several studies of invasive BCIs with functional electrical stimulation (FES) of muscles were conducted.

First, researchers demonstrated the ability of a patient with long-term tetraplegia to control certain movements of a virtual arm by using a BCI and simulating the work of specific muscles. For motor simulation, the estimated parameters of muscle contractile force and arm weight were considered [82].

In subsequent FES studies, the researchers succeeded in achieving BCI-controlled functional arm movements [83, 27]. In the first of them, a participant with C5–C6 SCI and over four year tetraplegia managed to get control of six different movements of his own wrist and hand, and was also able to take a bottle, pour its contents into a glass, put the bottle down, and mix the contents of the glass with a stirrer on average in 42 s [83]. In this study, machine learning algorithms were used to process neuronal signals. Electrical stimulation of the paralyzed muscles was conducted using 130 electrodes in a flexible sleeve, which was wrapped around the right forearm. The training continued for 15 months (up to three training sessions per week). The average control accuracy was 70%. Clinical assessment showed that with BCI-FES the patient’s motor abilities corresponded to the level of spinal cord lesion of the C7–T1 level, which is two vertebrae below the actual damage. This improvement is significant in reducing the burden of care for patients with C5–C6 SCI, as the majority of them require assistance with daily activities, whereas patients with C7–T1 SCI can live more independently.

In the next study, a patient with consequences of high SCI (C4 ASIA A) was able to successfully drink a cup of coffee and self-feed using BCI–FES [27]. The training was conducted for 18 weeks with an average of 8 h per week. The FES system included 36 transcutaneous electrodes to stimulate the muscles of the hand, forearm, and shoulder. The “drink coffee” task required a series of sequential actions: 1) straighten the elbow; 2) relax the grip; 3) take a cup; 4) bend the elbow to bring the cup to the mouth; 5) take a sip using a straw; 6) straighten the elbow to return the cup; 7) loosen the grip. These processes in total took from 20 to 40 s, and 11 out of 12 attempts were successful.

## Bidirectional brain–computer–brain interface

Bidirectional brain–computer–brain interface allows not only to record signals from the cerebral cortex, but also to modulate its activity. Such an interface additionally includes external tactile sensors and electrodes implanted in the somatosensory cortex. Sensory feedback is of key importance to the majority of motor tasks, providing information about the location of the limb, about touching an object, and characteristics of that object. Sensory and motor functions are not independent: the brain creates complex motor plans and compares the expected result with the sensory feedback in order to appropriately adjust the movement [84].

The advantages of bidirectional BCIs were demonstrated in [23]. A patient with C5–C6 SCI had two electrode arrays implanted into each of the motor and somatosensory cortex of the dominant hemisphere, and the touch and pressure sensors were built into the hand of the robotic arm in the BCI circuit. The patient first learned to control the robotic arm with the visua feedback only. Then, with the tactile feedback added, the quality and speed of the robotic hand movements improved within the first four sessions. This was quantitatively assessed using the ARAT test, the average score for which increased from 17 to 21, and the test completion rate decreased by over 2 times [23].

## High-performance communication neural interfaces

Due to continuous developments in the accuracy, speed, and stability of neural cursor control, patients with implanted cortical BCI sensors can type text with a speed sufficient for communication. However, recent advances in neural communication interfaces allow to type text or reproduce speech without using a virtual keyboard. Over the history of communication neural interfaces with intracortical sensors development (in 2000–2023), the speed of text reproduction increased from 3 characters per minute [61], which is comparable to the EEG–BCI performance, to 60 words per minute [24], which is approximately the speech rate of a healthy person (Table 2).

**Table 2:** Results of developments and clinical studies of communication neural interfaces with intracortical sensors

References	Patients	BCI features	Results
Kennedy et al., 2000 [61]	M., 53 years, brainstem stroke which occurred 3 months before the experiment, 2–17 months after implantation	1 (neurotrophic) implant in the anterior central gyrus, cursor control, paradigm: imagining of movement, then voluntary control	Typing — 3 characters per minute
Guenther et al., 2009 [64]	M., 26 years, brainstem stroke	1 (neurotrophic) implant in the speech articulation area on the border of M1 and the premotor cortex of the left hemisphere, control of the speech synthesizer during the attempts to pronounce sounds	Reproduction of vowel sounds with accuracy of 70%
Bacher et al., 2015 [85]	F., 58 years, brainstem stroke which occurred 15 years before the experiment, 1589–1925 days after implantation (S3)	1 implant in the anterior central gyrus, cursor control, comparison of the radial and QWERTY keyboard layouts	With radial layout, typing speed — 10 CSM, accuracy — 92%; internet chat: 8.1 CSM, accuracy — 100%
Jarosiewicz et al., 2015 [86]	1) F., 58 years, brainstem stroke, 5 years after implantation (S3); 2) M., 66 years, brainstem stroke, 4 months after implantation (T2); 3) F., 51 years, ALS, 10 months after implantation (T6); 4) M., 58 years, ALS, 6 months after implantation (T7)	1–2 implants in the anterior central gyrus, optimized signal processing algorithms	Typing speed 10–22 CSM remained 2 h within several days without the need for additional calibration
Gilja et al., 2015 [79]	1) F., 51 years, ALS, 151–628 days after implantation (T6); 2) M., 54 years, ALS, 349–387 days after implantation (T7)	1 (T6) or 2 (T7) implants in the anterior central gyrus, optimized signal processing algorithms and experimental design	Typing speed — 34 characters (6 words) per minute
Pandarinath et al., 2017 [87]	1) F., 52 years, ALS, 570–621 days after implantation (T6); 2) M., 54 years, ALS, 537–548 days after implantation (T7); 3) M., 63 years, SCI C4 ASIA C which occurred 9 years before the experiment, 55–70 days after implantation (T5)	1 (T6) or 2 (T5 and T7) implants in the anterior central gyrus, optimized signal processing algorithms	Average typing speed: T6 — 32 CSM (6 words per minute), T7 — 13.5 CSM (3 words per minute), T5 — 39 CSM (8 words per minute) Max. speed: T6 — 40 CSM, T7 — 29.5 CSM, T5 — 40.5 CSM
Nuyujukian et al., 2018 [88]	1) F., 53 years, ALS, 1013–1034 days after implantation (T6); 2) M., 51 years, ALS, 218–225 days after implantation (T9); 3) M., 63 years, SCI C4 ASIA C, 121–140 days after implantation (T5)	1 (T6) or 2 (T5 and T9) implants in the anterior central gyrus, signal transmission via Bluetooth to control a tablet (e-mail, chat)	Average typing speed: T6 — 24 CSM, T9 — 14 CSM, T5 — 31 CSM Max. speed (w/o autocorrection): T6 — 33 CSM, T7 — 15.5 CSM, T5 — 40 CSM
Simeral et al., 2021 [89]	1) M., 65 years, SCI C4 ASIA C, 560–588 days after implantation (T5); 2) M., 35 years, SCI C4 AIS-A, 307–361 days after implantation (T10)	2 implants in the anterior central gyrus (T5) or 1 implant in the anterior central and 1 implant in the medial frontal gyrus, wireless BCI system for home use	Control accuracy: 98% (T5) and 95% (T10), typing speed — 13.4 CSM (T5) Reliable signal transmission was possible when recording within 24 h
Willett et al., 2021 [22]	M., 68 years, SCI C4 ASIA C, 1211–1239 days after implantation (T5)	2 implants in the anterior central gyrus, control paradigm: imagining of the letters writing with a pen	Typing speed — 90 characters (18 words) per minute accuracy w/o autocorrection — 94%, with autocorrection — 99%
Shan et al., 2023 [90]	M., 70 years, SCI C4 ASIA C (T5)	2 implants in the anterior central gyrus, control paradigm: imagining of finger typing on a keyboard with a specific layout	Typing speed — 14 CSM (potentially — 26 CSM), control accuracy — 90%
Willett et al., 2023 [24]	Patient with bulbar ALS (T12)	2 implants in the ventral premotor cortex and 2 in the Broca’s area, control paradigm: imagining of words pronunciation (articulation)	Speech rate: 62 words per minute, accuracy of words recognition — 88% for 125,000 words vocabulary

Note: SCI — spinal cord injury; ALS — amyotrophic lateral sclerosis; S3, T2, T5, T6, T7, T9, T10, T12 — patient IDs in the BrainGate project; M./F. — patient’s gender; CSM — correct symbols per minute; M1 — primary motor cortex.

At first, the keyboard layout was optimized to improve the performance of communication BCIs for the neural cursor control. The radial layout allowed to repeat text in the copy task at an average speed of 10 correct characters per minute and an accuracy of over 90% [85]. Keyboard layout optimization increased typing accuracy by 37–65%. These indicators were achieved by a patient with a 14-year history of anarthria and tetraplegia during three training sessions and a sensor implanted about 5 years before the experiment [85].

In further studies, optimization of approaches to signal processing allowed to achieve more stable control quality without the need for regular calibration [86] and increase the average typing speed to 39 correct characters (8 words) per minute; the maximum speed was 40.5 correct characters (9 words) per minute without auto-correction [79, 87]. The achieved typing speed was inferior to the communication performance of a healthy person in normal conditions: the typing speed on a smartphone is 115 characters (12–19 words) per minute, and the speech rate is 90–170 words per minute. However, the achieved speed of typing and cursor control allowed several paralyzed patients to use a tablet with a standard user interface to communicate in emails and chats, make search queries and use basic applications [88].

Further performance improvement of communication BCIs was achieved by applying a totally different paradigm for signal control. A patient paralyzed due to SCI imagined that he was writing words with a pen. The BCI system successfully learnt to recognize each letter, and the typing speed reached 90 characters (18 words) per minute with an accuracy of 94% in real time or >99% with autocorrection [22]. The paradigm of imagining the text-writing procedure turned out to be essentially simpler for the signal decoding than controlling the cursor movement to select letters. Researchers [22] believe that this is due to the fact that handwritten letters are easier to be distinguished than point-to-point movements as the spatiotemporal patterns of the letters neural activity vary more than straight line movements.

Application of another paradigm option — imagining typing with fingers on a virtual keyboard with a special symbol layout — allowed to achieve control accuracy of 95%. However, in terms of typing speed (14 characters per minute), this approach was inferior to typing by the neural cursor movement or using the imagining writing letters with a pen [90].

The latest publication to date, which is dedicated to neural communication interfaces with intracortical sensors, reports the development of a high-performance speech neural prosthesis [24]. A patient with ALS, who was unable to intelligibly speak, had 2 microelectrode arrays implanted in the Broca’s area and 2 in the ventral premotor cortex of the dominant hemisphere. The patient’s attempt to speak was interpreted by the BCI system at a speech rate of 62 words per minute, which was close to normal speech rate. After improvement of the language model used for word recognition, the error rate was 12% for the vocabulary of 125,000 words. This was the first successful demonstration of a large word stock interpretation using neurotechnology. The source of a reliable signal, contrary to classical approaches, was not the Broca’s area (area 44), but the ventral premotor cortex (area v6). Based on the results of this study, the researchers identified two aspects of the neural code of speech, which were promising for the speech BCIs and were preserved years after the onset of paralysis: spatially-varied adjustment to speech articulators, which allowed accurate signal decoding from a small area of the cerebral cortex, and detailed articulatory representation of phonemes.

## Conclusion

In recent years, one can see a continuous development of invasive neural interface technologies. However, while the development and research related to non-invasive BCIs is conducted in many countries, clinical studies of invasive BCIs, due to the need for significant funding, can only be conducted by a few research groups in the world. Each year their data enrich our understanding of the brain functioning and provide new capabilities for rehabilitation of patients with severe functional impairment. Such interdisciplinary achievements and research of invasive BCIs contribute to significant progress in development of both neurobiology and information technology.

Further advances here will be related to the increase of speed, accuracy, and multi-task BCI control by improving the design of neural implants and their biocompatibility; development of methods for obtaining biosignals and improvement of algorithms for their extraction and decoding [16, 91–100]; adjustment of the BCI design for home use [88, 89]. Also, BCI technologies are developed to restore locomotion [48] and use in pediatrics [101]; visual and auditory bionic prostheses are being designed [102]. Due to a gradual integration of BCI technologies into clinical practice, issues of standardization and bioethics shall unavoidably arise [103].
